# The Clinical Significance of Subtle Distal Fallopian Tube Abnormalities: A Multicentre Prospective Observational Study

**DOI:** 10.52054/FVVO.16.1.007

**Published:** 2024-03-28

**Authors:** X Zheng, X Yu, X Xie, G Lyu, J Niu, X Li, H Chen, A Watrelot, J Guan

**Affiliations:** Reproductive Medicine Center, Department of Obstetrics and Gynaecology, Peking University People’s Hospital; Reproductive Medicine Center, Linyi People’s Hospital; Department of Gynaecology, Shenyang Women’s and Children’s Hospital; Department of Gynaecology, Obstetrics & Gynaecology Hospital of Fudan University; Lyon Natecia Hospital

**Keywords:** Fimbrial agglutination, tubal diverticula, tubal accessory ostium, fimbrial phimosis, accessory fallopian tube, Hydatid of Morgagni

## Abstract

**Background:**

Subtle distal fallopian tube abnormalities are a group of diseases characterised by small variations in tubal anatomy. The clinical significance of these abnormalities need to be studied.

**Objectives:**

The purpose of this multicentre prospective observational study was to investigate whether subtle distal fallopian tube abnormalities are related to infertility and endometriosis.

**Materials and Methods:**

The investigation was carried out in five medical centres in China and France from February to July 2021 and included reproductive-age patients who underwent gynaecological laparoscopy. Subtle abnormalities included Hydatid of Morgagni (HM) , fimbrial agglutination, tubal diverticula, accessory ostium, fimbrial phimosis, and accessory fallopian tube.

**Results:**

642 patients were enrolled in the study and 257 (40.0%) were diagnosed with subtle tube abnormalities. Hydatid of Morgagni was the most common abnormality (22.7%; n=146), followed by fimbrial agglutination (19.8%; n=127), tubal diverticula (6.9%; n=44), accessory tube (2.0%; n=13), and tubal accessory ostium (1.9%; n=12). Fimbrial phimosis was the least common abnormality (0.3%; n=2). The prevalence of subtle fallopian tube abnormalities was significantly higher among infertile patients (188/375, 50.1%) than those without history of infertility (69/267, 25.8%, ᶍ2=38.332, P=0.000). 209 patients were diagnosed with endometriosis during surgery, and the prevalence of subtle abnormalities was significantly higher in the endometriosis group than in those without endometriosis (61.2%, [128/209] vs. 29.8% [129/433], ᶍ2=58.086, P=0.000).

**Conclusions:**

Higher prevalence of subtle tubal abnormalities suggests that they may contribute to infertility. They are highly related to endometriosis and indicate fimbrial abnormalities of endometriosis.

**What is new?:**

This is the largest multicentre study to investigate the subtle distal fallopian tube abnormalities in infertile women. Compared to previous studies, this study includes the main subtle distal abnormalities and the control group patients without a history of infertility.

## Introduction

Subtle distal fallopian tube abnormalities are a group of diseases characterised by subtle variations in tubal anatomy, including Hydatid of Morgagni (HM), fimbrial agglutination, and tubal diverticula (Guan and Watrelot, 2019). Although this group of tubal diseases was introduced in the literature more than a century ago, they were thought for a long time to be normal anatomic variations and not clinically significant. Some studies have reported that these abnormalities may contribute to female infertility, but the value of these studies was undermined by the small number of study participants and only included one or two types of abnormalities (Cohen, 1987; Yablonski et al., 1990; Han et al., 2014;Zheng et al., 2015).

Fakih and Marshall (1994) first reported that subtle distal fallopian tube abnormalities are common in the infertile population with pelvic endometriosis and can significantly lower the pregnancy rate. Our previous studies also supported that these abnormalities are present at high rates in the infertile population with endometriosis (Zheng et al., 2022; Zheng et al., 2023). However, no large study has compared the prevalence of these abnormalities in an infertile population and fertile control group. Furthermore, no study has investigated their relationship with endometriosis in the general reproductive-age population. The purpose of the present study was to investigate whether the prevalence of subtle distal fallopian tube abnormalities is higher in an infertile population than in the general population and its relationship with endometriosis.

## Materials and Methods

### Patients and study design

This was a prospective multicentre observational study conducted from February to July 2021. Five medical centres participated in this study: Peking University People’s Hospital (Beijing, China), Lyon Natecia Hospital (Lyon, France), Shenyang Women’s and Children’s Hospital (Shenyang, China), Linyi People’s Hospital (Linyi, China) and Obstetrics & Gynaecology Hospital of Fudan University (Shanghai, China). The study was registered with the Chinese Clinical Trial Registry (reference: ChiCTR2000029095) and approved by the Human Research Ethics Committee of Peking University People’s Hospital.

The study population consisted of reproductive- age patients who were admitted to undergo gynaecological laparoscopy. The inclusion criteria were patient age between 18 and 45 years and indication for gynaecological laparoscopy. The exclusion criteria were age <18 or >45 years, acute pelvic inflammatory disease, frozen pelvis or gross distortion of the tubal anatomy and gynaecological malignancy. All of the participants signed an informed consent form to participate in this study.

### Definition of subtle distal fallopian tube abnormality

Six types of subtle distal fallopian tube abnormalities were included in the research: fimbrial agglutination, tubal diverticula, tubal accessory ostium, fimbrial phimosis, accessory fallopian tube, and Hydatid of Morgagni. If fimbrial abnormalities were identified, they were classified as either unilateral or bilateral. More than one fimbrial abnormality could be found in the same fallopian tube.

**Figure 1:**
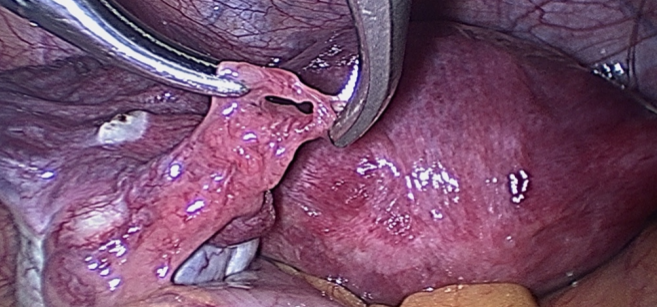
Fimbrial agglutination.

**Figure 2:**
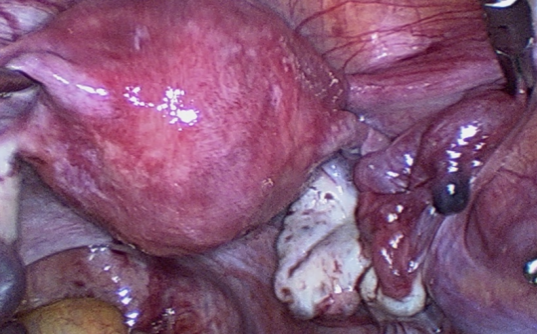
Tubal diverticula.

**Figure 3:**
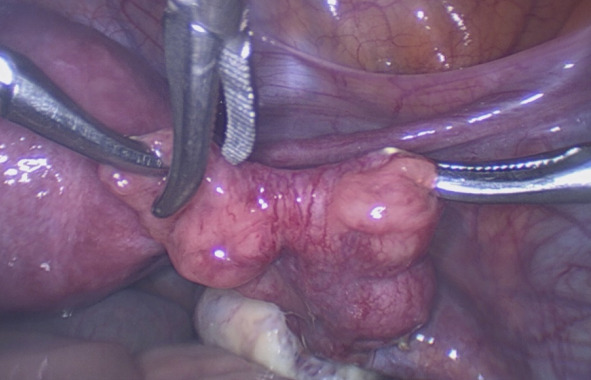
Tube accessory ostium.

**Figure 4:**
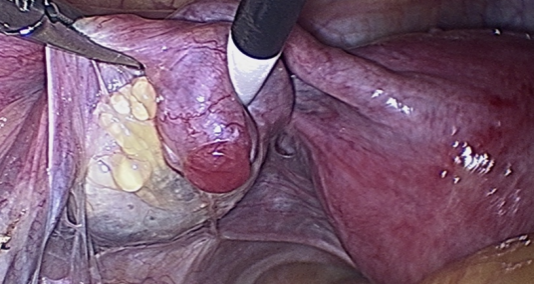
Tubal phimosis.

**Figure 5:**
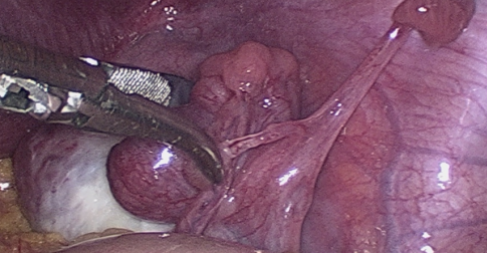
Accessory fallopian tube.

**Figure 6:**
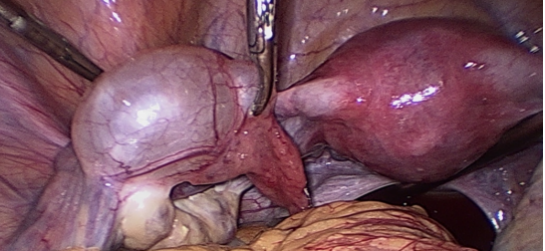
Morgagni hydatids.

Fimbrial agglutination (Figure 1) is defined as one or more adhesive bridges of fimbria across the ostium. Tubal diverticula (Figure 2) is defined as a thin-walled, outpouching, clear cyst in the ampullary or fimbrial portion of the fallopian tube. Tubal accessory ostium (Figure 3) is defined as an ectopic fimbria located a distance from the fimbriated end. Fimbrial phimosis (Figure 4) is a concentric stricture located at the infundibular fimbrial junction of the fallopian tube. An accessory fallopian tube (Figure 5) is a congenital anomaly that is attached to the ampullary part of the main tube; the fimbrial end is open and has fine fimbriae lined with normal epithelium, similar to the main tube but with no communication between the lumen of the main tube. HMs (Figure 6) are small pedunculated structures attached to the fallopian tubes near their fimbriated end.

### Surgical procedure and follow-up

Laparoscopies were performed by senior gynaecological or reproductive surgeons familiar with the diagnostic criteria of subtle fallopian tube abnormalities. Laparoscopy was performed under general endotracheal anaesthesia. A systematic laparoscopic evaluation was completed to accurately evaluate the pelvic organs. Tubal patency was evaluated by the perfusion of diluted methylene blue dye if the patient was diagnosed with infertility according to World Health Organization (2018) criteria. Endometriosis was staged according to the Revised American Fertility Society Classification of Endometriosis (rAFS).

The surgical findings were recorded in a detailed electronic case report form for each patient. If the surgeons were uncertain about the diagnosis of subtle distal fallopian tube abnormalities, the surgery images or video were sent to the senior authors (JG or AW) for verification of the final diagnosis.

### Statistical analysis

All statistical analyses were performed using SPSS version 19.0 (SPSS Inc., Chicago, IL, USA). Data were statistically described in terms of means or frequencies (percentage) when appropriate. Numerical variables were compared by t tests and categorical data by Pearson’s chi-squared. Significance was defined as an alpha value of p < 0.05.

## Results

From February 2021 to July 2021, 642 patients who underwent laparoscopy at the participating centre were recruited into this study. The mean participant age was 31.72 ± 5.58 years. A total of 514 patients were Asian, 115 patients were Caucasian and the remaining 13 patients were African. The indications for surgery are listed in Table I. The most common indications for laparoscopy were tubal obstruction, endometriosis, and unexplained infertility. A total of 375 patients were diagnosed with infertility and 267 patients had no history of infertility.

**Table I:** Indications for surgery.

Surgery indication	No. of patients
Tubal obstruction diagnosed by hysterosalpingography (HSG)	238
Endometriosis (endometrioma diagnosed by ultrasound or positive findings of physical examination, such as painful nodule of the posterior cul-de-sac)	104
Unexplained infertility	91
Uterine fibroid	62
Ectopic pregnancy	60
Ovarian cyst	56
Adenomyosis	16
Caesarean scar pregnancy (CSP)	5
Recurrent implantation failure (RIF)	4
Chronic pelvic pain	5
Laparoscopic abdominal cerclage for cervical incompetence	4
Tubal sterilisation	1
Rudimentary horn of the uterus	1

Among these 642 cases, 257 (40.0%) were diagnosed with subtle fallopian tube abnormalities. The analysis of individual abnormalities revealed that HM composed the largest group (22.7%; n=146), followed by fimbrial agglutination (16.7%; n=107), tubal diverticula (7.0%; n=45), accessory tube (2.2%; n=14), and tubal accessory ostium (1.9%; n=12). Fimbrial phimosis was the least common abnormality (0.3%; n=2). In the 375 infertile patients, 188 (50.1%) were diagnosed with subtle fallopian tube abnormalities, which was a significantly greater proportion than the control group without a history of infertility (69/267, 25.8%, ᶍ2=38.332, P=0.000). Individual abnormality analysis shows the prevalence of HM, fimbrial agglutination, accessory ostium and fimbrial phimosis in the infertile population is higher than the the control group without a history of infertility. Individual abnormality characteristics are shown in Table II. Some patients had more than one type of abnormality.

**Table II:** Prevalence of individual subtle tubal abnormalities in women with and without endometriosis and history of infertility (Numbers in brackets are percentages, there may be more than one subtle abnormality in the same fallopian tube).

	Infertility		Endometriosis	
	Yes n=375	No n=267	Yes n=209	No n=433
Morgagni hydatids	108(28.8)*	38(14.2)	68(32.5)*	78(18.0)
Fimbrial agglutination	91(24.3)*	16(6.0)	58(27.8)*	49(11.3)
Tubal diverticula	25(6.7)	20(7.5)	23(11.0)*	22(5.1)
Accessory tube	10(2.7)	4(1.5)	9(4.3)*	5(1.2)
Accessory ostium	11(2.9)*	1(0.4)	10(4.8)*	2(0.5)
Fimbrial phimosis	2(0.5)*	0	2(1.0)*	0

* means significant difference between two groups ( p < 0.05).

In this cohort, 209 patients were diagnosed with endometriosis during surgery, and the prevalence of subtle abnormalities was significantly higher in the endometriosis group than in the non- endometriosis group (61.2% [128/209] vs. 29.8% (129/433), ᶍ2=58.086, P=0.000). Individual abnormality analysis shows the prevalence of all types of abnormalities in the endometriosis group were higher than in the non-endometriosis group.

The difference in prevalence of subtle abnormalities in the different ethnic groups was also investigated. The prevalence was 38.7% (199/514) among the Asian patients, 47.8% (55/115) among the Caucasian patients and 23.1% (3/13) among the African patients. There was no significant difference seen between the different ethnic groups (ᶍ2=4.838, P=0.089).

## Discussion

The fallopian tubes play an important role in sperm transport, oocyte capture and transport, fertilisation and early embryo development. Abnormalities of the fallopian tube can impair tubal peristaltic or ciliary activity and impair the function of gamete transport and fertilisation, causing infertility and ectopic tubal pregnancy.

Subtle distal fallopian tube abnormalities were first reported more than a century ago. Watson (1902) described the definition, classification and pathological characteristics of tubal Hydatid of Morgagni. In another article, Gardner et al. (1948) introduced the embryonic origin and definition of an accessory tube and tubal HM. For a long time, subtle distal fallopian tube abnormalities were regarded as normal variations of the fallopian tube and to be of little clinical significance.

However, tubal disease accounts for 25- 35% of female factor infertility, with more than half of cases being due to salpingitis. Proximal tubal blockage and hydrosalpinx have been well investigated and systematic therapy choices have been proposed. In contrast, subtle distal fallopian tube abnormalities have received negligible attention regarding their role in infertility. Yablonski et al. (1990) reported that fimbrial agglutinations, accessory tubes, accessory ostia, phimosis and sacculations were more common in infertile women than in fertile women at cesarean section. The authors suggested that they contribute to infertility. The limitation of this study was that the infertile group underwent laparoscopy and the control group underwent cesarean section during delivery. Subtle distal fallopian tube abnormalities are tiny abnormalities that would be missed during open surgery, which may cause an underestimation of the true prevalence in the fertile population. Other studies reported that the prevalence of subtle distal tubal disease in the infertile population was 2-10% (Cohen, 1987; Han et al., 2014; Zheng et al., 2015). These studies were small-scale case reports and focused on only one type of disease. In our recent single-centre cohort study, the prevalence of five types of subtle distal fallopian tube abnormalities (fimbrial agglutination, tubal diverticula, accessory ostium, fimbrial phimosis, and accessory fallopian tube) in the infertile population was 28.65%. The limitations of that study were a lack of control group and the exclusion of tubal HM (Zheng et al., 2021).

Considering the limitations of previous studies, we designed this multicentre observational study to better evaluate the relationship between subtle distal fallopian tube abnormalities, and infertility and endometriosis. All of the participating centres have a vast clinical experience in diagnosing subtle tubal abnormalities to ensure that the fallopian tubes were carefully investigated during laparoscopy. The prevalence of subtle distal fallopian tube abnormalities was 50.1% in the infertile population, which is significantly higher than the prevalence in the control group without a history of infertility (25.8%), especially the HM, fimbrial agglutination, accessory ostium and fimbrial phimosis, indicating that they are highly related to female infertility.

How subtle distal fallopian tube abnormalities induce infertility remains unclear, but several mechanisms could contribute to pathogenesis. First, tubal abnormalities could hinder oocyte capture, transport, and fertilisation. The adhesive bridges of the fimbrial ostium of fimbrial agglutination could interfere with ovum pick-up. An abnormal weight of the HM in the distal part of the tube may disrupt tubal motility and ovum pick-up by the fimbriae (Cebesoy et al., 2010). One possible mechanism of the accessory ostium is that the ovum can escape from the tube through the accessory ostium (Zheng et al., 2015). Secondly, concurrent endometriosis is also an important factor contributing to infertility. Thirdly, uterine receptivity is affected. Savaris et al. (2006) reported that the expression of the b3 integrin subunit is significantly reduced in tubal phimosis patients during the window of implantation.

Endometriosis is a well-known cause of female infertility. The pathophysiological mechanisms of endometriosis-related infertility are distorted pelvic anatomy, altered peritoneal function, endocrine and ovulatory function, impaired implantation and diminished oocyte and embryo quality. Most physicians are aware that stage III and IV endometriosis can cause severe peri-tubal adhesions and tubal occlusion. Little is known about the relationship between endometriosis and subtle tubal abnormalities.

Fakih and Marshall (1994) researched tubal abnormalities (including tubal sacculations, diverticula, convolutions, phimosis, fimbrial agglutination, and peri-tubal adhesions) in endometriosis patients and found that the prevalence of tubal abnormalities in mild, moderate and severe endometriosis was 30%, 42%, and 55%, respectively (Fakih and Marshall, 1994). The limitation of this study was that it included peri-tubal adhesions, which are not a subtle tubal abnormality. Abuzeid et al. (2007) reported a significantly higher prevalence of fimbrial pathology in the endometriosis group (50.2%) than in the non-endometriosis group (17.8%). However, this study excluded stage III-IV endometriosis. In our previous study on the prevalence of subtle tubal abnormalities in an infertile population, 44.6% of infertile endometriosis (178/399) patients had subtle tubal abnormalities (Zheng et al., 2023). In the present multicentre study, 61.2% of endometriosis (128/209) patients had subtle tubal abnormalities. The prevalence of all types of subtle tubal abnormalities in the endometriosis group were higher than in the non- endometriosis group. All of these studies support this group of diseases being highly related to endometriosis and indicate fimbrial abnormalities of endometriosis.

How endometriosis induces subtle tubal abnormalities is unclear, but several mechanisms could contribute to pathogenesis. Firstly, the origin of some subtle tubal abnormalities is congenital, arising from embryological remnants. Therefore, one could imagine that what is able to create severe adhesions and fibrotic nodules is also able to grow tiny, almost invisible embryological remnants derived from epoophoron, paroophoron, and Gartner duct cyst. In addition, certain inflammatory factors related to endometriosis may damage the tubal anatomy, causing subtle variations, including fimbrial agglutination and fimbrial phimosis (Suginami et al., 1988). The invasion of endometriosis lesions may damage the muscular layer of the tube, causing tubal diverticula. As the disease progresses, the endometriosis lesion may penetrate the serosa of the fallopian tube, causing the development of accessory ostia.

This study supports the theory that subtle tubal abnormalities are fimbrial abnormalities of endometriosis. However, this group of diseases has long been neglected by gynaecological surgeons, and even reproductive specialists, the likely reason being that they are non-occlusive tubal abnormalities. When chromopertubation is performed to evaluate tubal patency, most physicians only observe whether the methylene blue dye can spill out of the fimbria and subtle tubal abnormalities are often neglected. For this reason, most early reports of subtle tubal abnormalities when seen during a laparoscopic gamete intrafallopian transfer (GIFT) procedure (Fakih and Marshall, 1994; Isherwood et al., 1990). During GIFT, the fimbriae are grasped with fimbrial-grasping forceps before a catheter is passed into the tube, providing an opportunity for careful examination of the distal part of the tube.

Our study has some limitations. Firstly, the patients without a history of infertility did not undergo tubal chromopertubation. This may have caused missed diagnosis of some kinds of subtle tubal abnormalities, especially tubal diverticula and fimbrial phimosis, in the non-infertile population.

Secondly, fewer African females were enrolled in this study than Asian and Caucasian females. Even though it seems that African females are less likely to be affected by this disease, too few African female participants were included to observe a significant difference. To investigate the prevalence in the different ethnic groups, a study including a more diversified ethnic population will be needed.

## Conclusion

Higher prevalence of subtle tubal abnormalities suggests that they may contribute to infertility.A systematic investigation of the fallopian tube, especially the distal part where most lesions occur, should be performed in every infertile patient undergoing laparoscopy. This group of diseases is strongly related to endometriosis and identifies fimbrial abnormalities of endometriosis.
